# CRISPR/Cas9-Mediated Knock-Out of Kras^G12D^ Mutated Pancreatic Cancer Cell Lines

**DOI:** 10.3390/ijms20225706

**Published:** 2019-11-14

**Authors:** Eva Lentsch, Lifei Li, Susanne Pfeffer, Arif B. Ekici, Leila Taher, Christian Pilarsky, Robert Grützmann

**Affiliations:** 1 Department of Surgery, Friedrich-Alexander-Universität Erlangen-Nürnberg (FAU) and Universitätsklinikum Erlangen, 91054 Erlangen, Germany; eva.pilarsky@googlemail.com (E.L.); susanne.pfeffer@uk-erlangen.de (S.P.); 2 Division of Bioinformatics, Department of Biology, Friedrich-Alexander Universität Erlangen-Nürnberg (FAU), 91054 Erlangen, Germany; lilifeifiona@hotmail.com (L.L.); leila.taher@fau.de (L.T.); 3 Institute of Human Genetics, Friedrich-Alexander-Universität Erlangen-Nürnberg (FAU) and Universitätsklinikum Erlangen, 91054 Erlangen, Germany; arif.ekici@uk-erlangen.de

**Keywords:** CRISPR/Cas9 system, G12D, Kras, Suit-2, Panc-1, TB32047

## Abstract

In 90% of pancreatic ductal adenocarcinoma cases, genetic alteration of the proto-oncogene Kras has occurred, leading to uncontrolled proliferation of cancerous cells. Targeting Kras has proven to be difficult and the battle against pancreatic cancer is ongoing. A promising approach to combat cancer was the discovery of the clustered regularly interspaced short palindromic repeat (CRISPR)/CRISPR-associated (Cas) system, which can be used to genetically modify cells. To assess the potential of a CRISPR/CRISPR-associated protein 9 (Cas9) method to eliminate Kras mutations in cells, we aimed to knock-out the c.35G>A (p.G12D) Kras mutation. Therefore, three cell lines with a heterozygous Kras mutation (the human cell lines SUIT-2 and Panc-1 and the cell line TB32047 from a KPC mouse model) were used. After transfection, puromycin selection and single-cell cloning, proteins from two negative controls and five to seven clones were isolated to verify the knock-out and to analyze changes in key signal transduction proteins. Western blots showed a specific knock-out in the Kras^G12D^ protein, but wildtype Kras was expressed by all of the cells. Signal transduction analysis (for Erk, Akt, Stat3, AMPKα, and c-myc) revealed expression levels similar to the wildtype. The results described herein indicate that knocking-out the Kras^G12D^ mutation by CRISPR/Cas9 is possible. Additionally, under regular growth conditions, the knock-out clones resembled wildtype cells.

## 1. Introduction

Pancreatic cancer (PaCa) is the fourth leading cause of all cancer death in the United States of America and Germany. Smoking, chronic pancreatitis, heavy alcohol consumption or genetic predispositions, such as Lynch syndrome, are only a small variety of risk factors. Common symptoms, including weight loss, abdominal or back pain, mean that identification during the early stages can be difficult. With its disastrous five-year survival rate of about 8%, the chances of survival after diagnosis are remote. Besides chemotherapy or radiation therapy as therapeutic options to alleviate pain or extend survival, the only potentially curative prospect remains resection [1,2,3,4]. Therefore, a better understanding of the functions of the tumor is fundamental to develop new treatment strategies and identify new therapeutic targets.

In about 90% of PaCa cases, a Kras mutation is detectable [5]. Kras is a proto-oncogene first discovered and identified in Kirsten rat sarcoma virus. It is part of the G-protein family. In contrast to most heterotrimeric G proteins, Kras is monomeric. As a central element in multiple signaling pathways, it is involved in the regulation of cell proliferation, differentiation, and survival. A point mutation in the protein leads to impairment of guanosine triphosphate (GTP)ase activity and thus continuous transmission of non-existent growth signals [6]. Activated mutations in the proto-oncogene Kras are a hallmark of PaCa. Its activity leads to a major role in PaCa initiation but its importance in maintaining the tumor is uncertain [7,8,9].

In recent years, whole-genome sequencing led to the discovery of clustered regularly interspaced short palindromic repeat (CRISPR) sequences and CRISPR-associated (Cas) genes in various bacteria and archaea [10]. The identification of these sequences, resembling DNA from viruses or plasmids, suggests that it is a bacterial adaptive immune system that provides particular defense against viral intruders [11,12,13]. CRISPR-associated protein 9 (Cas9) is the most commonly used among the Cas proteins. Cas9-mediated cleavage of DNA operates with two RNAs: (i) CRISPR-RNA (crRNA), which recognizes foreign DNA through a complementary region 20-base pairs (bps) in length, called the proto-spacer adjacent motif (PAM), and (ii) trans-acting CRISPR-RNA (tracrRNA), which hybridizes with the crRNA [14,15]. To facilitate this system, the crRNA–tracrRNA fusion can be combined into a chimeric single-guide RNA (sgRNA) [16]. By creating a 20-nucleotide region complementary to a DNA sequence of interest in the sgRNA, Cas9 can be aimed at any genomic locus with a suitable PAM sequence. After DNA tracking and cleavage, repair mechanisms can provoke insertion and deletion mutations (indels), which may cause a knock-out [17]. With CRISPR/Cas9 providing a new opportunity for modifying genes in general and the armed with the knowledge of overexpression of Kras in PaCa, the possible knock-out of mutated Kras was investigated.

## 2. Results

### 2.1. Expression of Kras^G12D^ and Total Ras after CRISPR/Cas9-Mediated Knock-Out in PaCa Cell Lines

**Figure 1:**
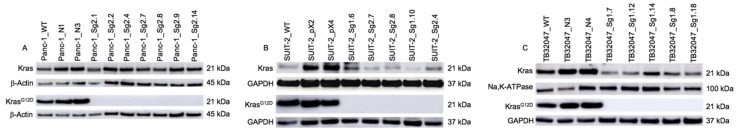
Kras expression by wildtype cells, negative controls and clones in Panc-1, SUIT-2 and TB32047 cell lines. (**A**) Western blots (WBs) showed similar bands in Panc-1. (**B**) SUIT-2 negative controls (N; pX) expressed more Kras protein than wildtype (WT) cells and clones. (**C**) TB32047 clones produced slightly less Kras protein than their WT and NC. No protein expression, indicating Kras^G12D^ knock-out, could be observed in Panc-1, SUIT-2 and TB32047 cell lines. β-actin, glyceraldehyde 3-phosphate dehydrogenase (GAPDH) served as loading controls.

To verify the role of mutant Kras in pancreatic cancer cell lines, two human (Panc-1 and SUIT-2) and a murine cell line (TB32047) were used to perform a CRISPR/Cas9-based knock-out of the mutated allele. All cell lines contained a heterozygous KRAS^G12D^ (c.35G>A) mutation. After transfection, puromycin selection and single-cell cloning, we produced about five to seven clones (Kras clones). Western blotting was performed to detect the knock-out effects introduced with the CRISPR/Cas9 gene editing system. In all tested cell lines, the results showed a knock-out with no protein expression of mutant protein Kras^G12D^. However, a normal total Kras protein expression level was observed for Panc-1 wildtype (WT) cell lines, negative controls (transfected with nonsense sgRNA molecules; NCs) and Kras clones. In the SUIT-2 cell line, WT and Kras clones resembled each other, whereas the protein expression in the negative controls was higher. Total Kras expression in the TB32047 clones was lower than in the WT and negative controls (Figure 1).

### 2.2. DNA Sequencing from Knocked-Out Clones

DNA sequencing was performed on all knock-out clones to confirm the presence of the target mutation in the sequence of Kras^G12D^. All knock-out clones had indel mutations in the sequence of Kras^G12D^. Most clones (Panc-1 2.1, 2.2, 2.8 and 2.9; SUIT-2 1.6, 2.7, 2.8, and 1.10; TB32047 1.7, 1.12, 1.14, and 1.18) had small indels (< 20 bp), while Panc-1 clones 2.7 and 2.14 had single-nucleotide insertions. Only three clones, one from each cell line (Panc-1 2.4, SUIT-2 2.4 and TB32047 1.8), had large indels (>20 bp) (see Table 1).

### 2.3. Expression of Key Signal Transduction Proteins in CRISPR/Cas9-Edited PaCa Cell Lines

**Figure 2:**
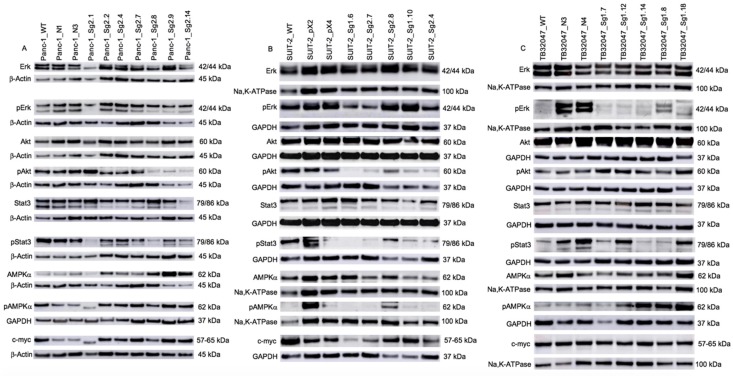
Expression of Erk, Akt, Stat3, AMPKα and c-myc proteins by wildtype cells, negative controls and Kras clones in Panc-1, SUIT-2 and TB32047 cell lines. Here, the prefix “p” indicates the phosphorylated version of the protein. (**A**) Panc-1 2.1, 2.8 and 2.14 expressed slightly less Erk than the others. Clone 2.1 also produced less pErk. Akt expression in Panc-1 showed no difference between WT, NC and clones. pAkt was reduced in Panc-1 clones 2.8, 2.9 and 2.14. Stat3 was expressed by all Panc-1 clones, except 2.14. Panc-1 showed reduced pStat3 expression in clone 2.1, 2.8 and 2.14. Lower AMPKα expression could be observed in Panc-1 WT, NC and clone 2.1. The pAMPKα expression level was lower in clone 2.1 than the other samples. Panc-1 NC and 2.1 produced less c-myc compared to the others. (**B**) SUIT-2 clones expressed Erk similar to the wildtype cells. SUIT-2 clones 1.6 and 2.7 produced less pErk. Akt expression in SUIT-2 cells showed no difference in expression between WT, NC and clones. Only WT cells, NC and clone 2.8 expressed pAkt. Clone 1.10 produced decreased Stat3 levels. pStat3 was only produced by SUIT-2 WT, pX2 and clone 2.6. Lower AMPKα expression could be observed in clones 1.10 and 2.4 compared to the WT cells. Just SUIT-2 pX2 and clone 2.6 produced pAMPKα. SUIT-2 clones 1.6, 2.7 and 2.4 produced less c-myc compared to the others. (**C**) Erk expression in TB32047 clones was similar to the wildtype. pErk was only expressed by TB32047 NC. Akt, pAkt, Stat3, AMPKα and c-myc protein levels were similar throughout all TB32047 samples. pStat3 was only produced by TB32047 NC, clones 1.12 and 1.18. TB32047 clones 1.12, 1.14, 1.8 and 1.18 expressed higher pAMPKα than the remaining samples. β-Actin, GAPDH and Na,K-ATPase served as loading controls.

After confirming the Kras^G12D^ protein knock-out, the effect on key signal transduction pathways was analyzed. Specifically, the (non-)phosphorylated proteins Erk, Akt, Stat3, AMPKα and c-myc were evaluated (Figure 2). We observed a heterogeneous expression pattern. Within Panc-1 cell clones pErk levels remained comparatively stable, however pAkt, pStat3, pAMPKα and c-myc levels were reduced in some Kras clones. This is contrary to the findings from the other cell lines. In the human cell line SUIT-2, we observed a loss of Akt phosphorylation while Erk phosphorylation was retained. pStat3 and pAMPKα proteins were only produced by clone 2.6 and c-myc levels were reduced in some Kras clones. However, within the murine KPC cell line TB32047, we found that the pErk levels and the phosphorylation of Akt and c-myc remained constant, the pStat3 protein was only produced by two clones, and most of the clones expressed pAMPKα.

### 2.4. TB32047 RNA Sequencing Results

**Figure 3:**
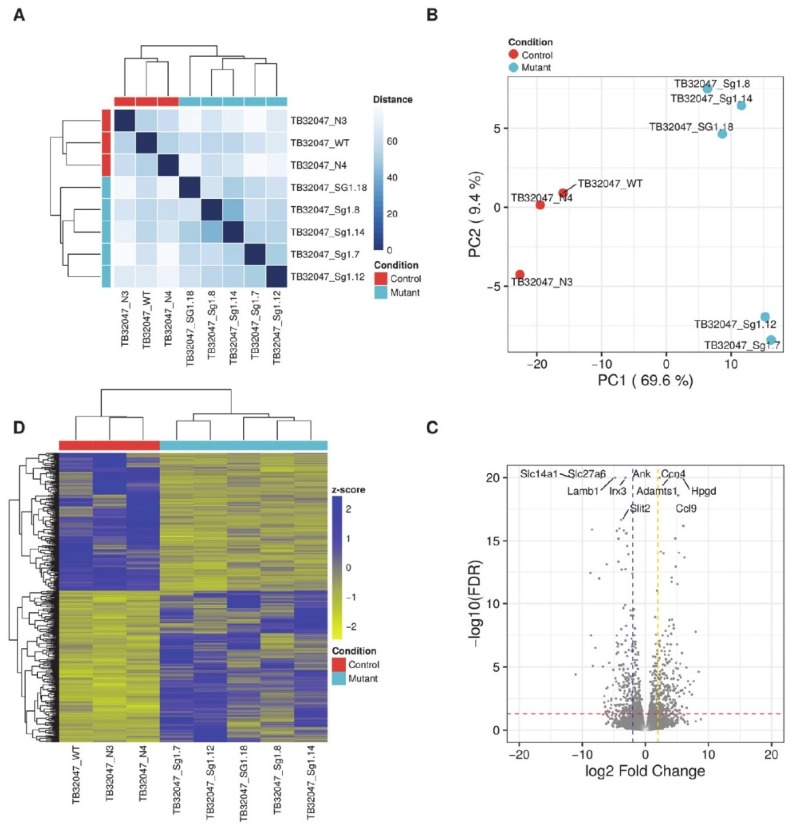
Differential expression analysis of edited TB32047 genes. (**A**) Sample-to-sample distances based on cell whole transcriptomes. The colors in the heatmap represent the Euclidean distances between pairs of samples, as calculated from the normalized rlog-transformed read counts of all genes. Samples were clustered using complete linkage. WT and NC samples grouped together and separated from Kras^G12D^ knock-out samples. (**B**) Principal component analysis (PCA) based on the normalized rlog-transformed read counts of differentially expressed genes (DEGs). PCA verified that most of the variance (69.9%, PC1) was associated with the altered expression between Kras^G12D^ knock-out samples and the WT and NC samples. (**C**) Volcano plot. Each dot represents a gene. The red horizontal line indicates a false discovery rate (FDR) of 0.05; the blue and yellow vertical lines highlight log2 fold-changes of −2 and 2, respectively. Up-regulated genes, about 218 DEGs, are located above the red line and right of the yellow line; down-regulated genes, about 199 DEGs, are located above the red line and left of the blue line. The 5 up- and down-regulated genes with the smallest FDR are labeled with their gene symbols. (**D**) Heatmap and hierarchical clustering of the samples and DEGs based on Euclidean distances between of normalized rlog-transformed counts. Rows have been centered and scaled to compute z-scores.

Since the edited TB32047 cells demonstrated some changes in the expression levels of key signal transduction proteins, we further investigated the differential gene expression of the CRISPR/Cas9 knock-out in this model system. RNA sequencing (RNA-seq) data from eight TB32047 samples (WT, N3, N4, sg1.7, sg1.8, sg1.12, sg1,14, and sg1.18) were analyzed to determine RNA expression changes in the Kras^G12D^ knock-outs. Based on their whole transcriptomes, the WT and NC samples grouped together and differed from the Kras^G12D^ knock-out samples (see Figure 3A). A total of 417 genes were differentially expressed between the two sample groups (see Supplementary data). Principal component analysis (PCA) based on the expression of the differentially expressed genes (DEGs) confirmed that most of the variance in the data (69.9%, PC1) was associated with expression changes between the Kras^G12D^ knock-out samples and the WT and NC samples (PC1, see Figure 3B). In addition, Kras^G12D^ knock-out samples were separated into two groups, although these differences were comparatively smaller (9.4% of the variance, PC2). Approximately half of the DEGs (218) were up-regulated, while the other half (199) were down-regulated (see Figure 3C). Among the down-regulated DEGs were *Lamb1* (log_2_ fold-change = −5) and *Slc27a6* (log_2_ fold-change = −7, Figure 3D). Functional and pathway analysis of the DEGs (see Supplementary Data) revealed that genes that were down-regulated in the Kras^G12D^ knock-out samples were enriched in biological processes, such as regulation of cell migration, differentiation, and proliferation, whereas genes that were up-regulated in the Kras^G12D^ knock-out samples were associated with inflammatory response, regulation of the ERK1/ERK2 cascade, and angiogenesis. Furthermore, up-regulated DEGs were enriched in the PI3K-Akt signaling pathway.

## 3. Discussion

As part of the G-protein family, Kras is involved in the regulation of cell proliferation and survival. Inactivation of its GTPase activity, as a result of mutation in the protein, leads to hyperactive effector signaling. Therefore, mutated Kras plays a major role in and is the driver of PaCa initiation. Mouse models have shown that an elevated frequency of activation in Kras leads to precursor lesions and the onset of PaCa [9,18,19]. However, if Kras is required for maintenance of PaCa remains to be elucidated.

We were able to successfully knock-out Kras^G12D^ using the CRISPR/Cas9 gene editing system in all of the three analyzed Kras heterozygous cell lines (Panc-1, SUIT-2 and TB32047). Interestingly, the cell cultivation showed no apoptosis or growth arrest, only a decreased growth rate compared to the wildtype could be observed. This could be due to knocking-out mutated Kras, which can result in a slower growth ability.

We verified the knock-outs using DNA sequencing and western blotting (WB). However, we observed no changes in the expression level of total Kras. These results suggest that Kras is not important for maintaining PaCa cells in vitro [9].

Furthermore, we looked for changes in key signaling pathways, such as mitogen-activated protein kinase (MAPK) or PI3K/Akt, which are known to be the major effector pathways of Kras activation. Our work indicates that the knock-out of Kras^G12D^ does not affect one common pathway of signal transduction, but that each cell line displays different effects. Others state that PI3K/Akt hyperactivation is important in PaCa cells lacking Kras [8]. But Panc-1 and TB32047 clones exhibited different expression patterns, while SUIT-2 clones were homogenously deficient in pAkt. The origin (human vs. mouse) could be one of the reasons why the outcome is different. Still, TB32047 cells are genetically engineered with a defined set of modification at Kras and p53.

If the clones are not using MAPK or PI3K/Akt pathway to survive, how can they resist apoptosis? Besides Erk and Akt, we analyzed the signal transduction proteins Stat3, AMPKα and c-myc. Stat3 has an anti-apoptotic effect induced by TGF-beta and is engaged in cell survival, whereas c-myc regulates cell cycle progression, cell growth, and apoptosis [20]. Our results showed a heterozygous expression of pStat3 among all clones from each cell line. In contrast, c-myc was stably expressed in Panc-1 and TB32047 clones. Some of the SUIT-2 clones expressed less c-myc than others. AMPKα influences metabolism by increasing catabolism and decreasing anabolism [21]. This is an important factor for cell survival, but expression of pAMPKα was hetergeneous just as pStat3. This leads to the assumption that every clone has evolved and should be considered individually.

Through RNA sequencing, we gained insight into the changed gene expression of edited TB32047 clones. We identified 417 differentially expressed genes. Two heavily down-regulated genes were Laminin B1 (*Lamb1*) and solute carrier family 27, member 6 (*Slc27a6*). As part of a subunit which assembles to a heterotrimeric isoform, Lamb1 facilitates cell differentiation, motility, adhesion and—as a ligand of the laminin receptor—it enables the ability of tumor cells to invade and build vessels [22]. Slc27a6 is member of a fatty acid transport protein family which uptakes long-chain fatty acids. This is important for energy metabolism and other cellular processes (i.e., signal transduction) [23]. After Kras^G12D^ knock-out these two necessary genes are down-regulated, which could negatively affect intensive angiogenesis, invasion and energy supply and therefore provide a useful tool to combat PaCa.

Nevertheless, Kapoor et al. showed that, after Kras^G12D^ repression in mice, pancreatic tumors can relapse. Kras^G12D^-independent tumors proliferated through Yap1/Tead2 activation. Therefore, overexpression of YAP1 may also promote growth in cells lacking Kras. This evasion could indicate that PaCa cells are able to grow using a different mechanism [24]. This correlation and the analysis of chemotherapy drug resistance are to be investigated in further experiments. Since confirming the possibility to knock-out Kras^G12D^, a next step could be to examine interactions between different pathways through taking aim at various targets in a multiplexed CRISPR/Cas reaction. sgRNA combinations can be used to increase knock-out efficiency [25].

## 4. Materials and Methods

### 4.1. Cell Culture

The human cell lines Panc-1 and SUIT-2 and the murine cell line TB32047 were used in this study. The Panc-1 cell line was bought from ATCC^®^ (American Type Culture Collection, Manassas, VA, USA; CRL-1469™, RRID:CVCL_0480). Panc-1 cells were cultured in Roswell Park Memorial Institute (RPMI) medium 1640 (ThermoFisher, Langenselbold, Germany; 21875-034). The SUIT-2 cell line was purchased from Japanese Collection of Research Bioresources Cell Bank (JCRB1094, RRID:CVCL_3172). SUIT-2 cells were cultured in minimum essential medium (MEM; ThermoFisher, Langenselbold Germany, 31095-029). The TB32047 cell line was obtained with courtesy from Prof. Dave Tuveson, Cold Spring Harbor Laboratory. TB32047 cells were cultured in Dulbecco’s modified Eagle’s medium (DMEM; ThermoFisher, Langenselbold Germany, 30966-021). Every culture medium was enriched with a final concentration of 10% fetal bovine serum (FBS) (ThermoFisher, Langenselbold Germany, A31608-01). All cell lines were grown under sterile conditions to avoid contamination. For cultivation the cells were incubated in monolayer culture at 37 °C, 5% CO_2_ and 80% air humidity. All cells were harvested by treatment with 0.25% trypsin-ethylenediaminetetraacetic acid (trypsin-EDTA; ThermoFisher, Langenselbold Germany, 25200-072).

### 4.2. CRISPR/Cas9 Gene Editing

In this study, Kras^G12D^ was knocked out by the CRISPR/Cas9 gene editing system. The following primers were synthesized by Eurofins Genomics: Hs_KRASm_sg1-forward (5′-CACCGTAGTTGGA GCTGATGGCGT-3′), Hs_KRASm_sg1-reverse (5′-AAACACGCCATCAGCTCCAATAC-3′), Hs_KRASm_sg2-forward (5′-CACCGCTTCTGGTAGTTGGAGCTGA-3′), Hs_KRASm_sg2-reverse (5′-AAACTCAGCTCCAACTACCACAAGC-3′). Cloning was performed using pSpCas9(BB)-2A- Puro (PX459) V2.0 vector, which was a gift from Feng Zhang (Addgene plasmid #62988; http://n2t.net/addgene:62988; RRID:Addgene_62988) [26]. The ligated vector was inserted into E.cloni^®^ 10G electrocompetent cells (BioCat, Heidelberg, Germany). Plasmid construction was performed according to protocol and confirmed by sequencing. The above-mentioned cell lines were transfected with Kras^G12D^ knock-out plasmid by Lipofectamine^®^ 3000 Transfection reagent (ThermoFisher, Langenselbold Germany; #L3000015) for 24 h. After one day the transfected cells were selected with puromycin (concentration 5 ng/μL). Mouse epidermal growth factor (mEGF) was added to the clone culture medium. Thereafter, single-cell cloning was performed in a 96-well plate to grow single clones. After growth, western blot and sequencing were performed to detect the knock-out.

### 4.3. Western Blot

To analyze the protein expression of the genetically modified cell clones, the cells were lysed using radioimmunoprecipitation assay (RIPA) buffer. The protein concentration was determined photometrically using Pierce^®^ BCA Protein Assay Kit. Gel electrophoresis was performed with Bolt™ 4–12% Bis-Tris Plus Gels (ThermoFisher, Langenselbold Germany, #NW04122BOX) and proteins were transferred to a nitrocellulose membrane. After blocking with 5% milk, the following primary antibodies were used: Akt (pan) (Cell Signaling Technology, Leiden, The Netherlands; #4691, RRID:AB_915783), phospho-Akt (Ser 473) (CST, #9271, RRID:AB_329825), AMPKα (CST, #5831, RRID:AB_10622186), phospho- AMPKα (Thr172) (CST, #2535, RRID:AB_331250), c-myc (CST, #5605, RRID:AB_1903938), p44/42 MAPK (Erk1/2) (CST, #4695, RRID:AB_390779), phospho-p44/42 MAPK (Erk1/2) (Thr202/Tyr204) (CST, #4370, RRID:AB_2315112), Ras (CST, #3339, RRID:AB_2269641), Ras (G12D Mutant Specific) (CST, #14429, RRID:AB_2728748), Stat3 (CST, #12640, RRID:AB_2629499), phospho-Stat3 (Tyr705) (CST, #9145, RRID:AB_2491009). β-Actin (CST, #8457, RRID:AB_10950489), glyceraldehyde 3-phosphate dehydrogenase (GAPDH; CST, #5174, RRID:AB_10622025) and Na,K-adenosine triphosphate (ATP)ase (CST, #3010, RRID:AB_2060983) served as loading controls. As secondary antibodies, horseradish peroxidase (HRP)-linked anti-mouse immunoglobulin G (IgG) (CST, #7076, RRID:AB_330924) and HRP-linked anti-rabbit IgG (CST, #7074, RRID:AB_2099233) were utilized. The signals were detected with an Amersham Imager 600 with SignalFire™ (Elite) ECL Reagent (Cell Signaling Technology, Leiden, The Netherlands, #6883 or #12757). All western blot assays were performed more than three times to validate the reliability of the results.

### 4.4. Confirmation of CRISPR/Cas9-Mediated Knock-out

To isolate DNA from the cell lines, NucleoSpin^®^ Tissue (MACHEREY-NAGEL, Düren, Germany) was used according to the manufacturer’s protocol. The DNA concentration was determined using the NanoDrop 2000 (ThermoFisher, Langenselbold Germany). PCR products for sequencing were amplified using Platinum™ SuperFi™ PCR Master Mix (ThermoFisher, Langenselbold Germany). PCR fragments were displayed using gel electrophoresis. The following primers were used for the human cell lines: hKras_f1 (AAGCGTCGATGGAGGAGTTT) and hKras_r1 (ACCCTCTCAC GAAACTCTGA); and for the murine cell line Mm_Kras_f1 (ACTCTGTACATCTGTAGTCACTG) and Mm_Kras_r1 (GCACGCAGACTGTAGAGCA) were used. PCR fragments were cloned into pMiniT 2.0 with the NEB^®^ PCR Cloning Kit (New England Biolabs, Frankfurt, Germany, E1202). For each single cell clone 10 bacteria colonies were picked and plasmid DNA was isolated (GeneJET Plasmid Miniprep Kit, K0503, ThermoFisher, Langenselbold Germany). Plasmid DNA was sequenced by Eurofins Genomics and the resulting sequences were aligned using Snapgene (GSL Biotech LLC; Chicago, IL, USA).

### 4.5. RNA Isolation and Preparation

The NucleoSpin^®^ RNA Plus kit (MACHEREY-NAGEL, Düren, Germany) was used to isolate RNA from the cell lines according to the manufacturer’s protocol. The RNA concentration and pureness were determined using a 2100 Bioanalyzer using the Agilent RNA 6000 Nano Kit (Agilent, Waldbronn, Germany; 5067-1511).

### 4.6. RNA Sequencing

Profiling of whole-genome transcriptomic patterns was performed with 500 ng of high-quality total RNA per sample by RNA sequencing at the next generation sequencing (NGS) core unit of the Medical Faculty of the FAU Erlangen. Libraries of the samples were generated using the Illumina stranded mRNA library kit. After sequencing on an Illumina HiSeq-2500 platform, the sequences were assigned to their sample of origin according to the used indices. The quality of reads was assessed using FastQC v0.11.4 [27]. Adapter sequences and stretches with low quality were removed using trimmomatic v0.36 [28] with the settings HEADCROP:13 CROP:85 LEADING:20 TRAILING:20 AVGQUAL:20 MINLEN:25. Reads were mapped to the mouse reference genome (Ensembl GRCm38) [29] using corresponding gene model annotation (v85) with STAR v2.5.3 [30] with parameters: --runThreadN 8 --genomeDir --readFilesIn --sjdbGTFfile --sjdbOverhang 100 --outSAMtype BAM SortedByCoordinate --outFileNamePrefix --quantMode TranscriptomeSAM GeneCount. Samtools v1.6 [31] was used to further process BAM files. Expression matrices were generated using RSEM v1.3.0 [32]. Differential expression analysis was performed for protein-coding genes using the R/Bioconductor package DESeq2 v1.16.1 [33] with standard parameters. WT, N3 and N4 were used as controls. Genes exhibiting a false discovery rate (FDR) ≤ 0.05 and a log_2_ fold-change ≥ 2 or ≤ −2 were considered differentially expressed. Normalized rlog-transformed gene counts were calculated using the DESeq2’s rlog() function.

### 4.7. Functional and Pathway Analysis

Functional and pathway analysis was performed with the Database for Annotation and Integrated Discovery (DAVID Bioinformatics Resources 6.8; http://david.abcc.ncifcrf.gov/) [34,35]. In particular, we focused on the ontologies: KEGG pathways, Biological Processes (BP), Molecular Function (MF) and Cellular Component (CC). Terms associated with a FDR ≤ 0.05 were considered statistical significantly enriched.

## 5. Conclusions

In conclusion, the CRISPR/Cas gene editing system can be used to knock-out Kras^G12D^ in human and mouse cell lines. This leads to cell clones that exhibit different aspects of Kras signal transduction.

## Figures and Tables

**Table 1:** Insertion and deletion mutation (indel) size of Panc-1, SUIT-2 and TB32047 clones. Indel size varies between single nucleotide indel, small: < 20 base pair (bp) and large > 20 bp inserts and/or deletions.

Panc-1 Clones	Indel Size	SUIT-2 Clones	Indel Size	TB32047 Clones	Indel Size
2.1	small	1.6	small	1.7	small
2.2	small	2.7	small	1.12	small
2.4	large	2.8	small	1.14	small
2.7	single nucleotide	1.10	small	1.8	large
2.8	small	2.4	large	1.18	small
2.9	small				
2.14	single nucleotide				

## Supplementary Materials

Supplementary materials can be found at https://www.mdpi.com/1422-0067/20/22/5706/s1.

## Abbreviations

bp	Base pair
Cas	CRISPR-associated
CRISPR	Clustered regularly interspaced short palindromic repeat
DEG(s)	Differentially expressed gene(s)
DMEM	Dulbecco’s modified Eagle’s medium
EDTA	Ethylenediaminetetraacetic acid
FBS	Fetal bovine serum
Indel	Inserts/deletions
MEM	Minimum essential media
NC	Negative control
PaCa	Pancreatic cancer
PDAC	Pancreatic ductal adenocarcinoma
PI3K	Phosphatidyl inositol 3-kinase
RIPA	Radioimmunoprecipitation assay
RPMI	Roswell Park Memorial Institute
seq	Sequencing
WB	Western blot
WT	Wildtype
