# Supplementary material for: CRISPR/Cas9-Mediated Knock-Out of KrasG12D Mutated Pancreatic Cancer Cell Lines
Source: Int J Mol Sci. 2019 Nov 14;20(22):5706. doi: 10.3390/ijms20225706 (PMC6888344; doi:10.3390/ijms20225706)
Supplement: Supplementary file 1 [file ijms-20-05706-s001.zip › Supplementary_Tables_Legends.docx]

**Supplemental Table Functional Analysis.** **Functional analysis of differentially expressed genes.** The mouse genome was used as background for the analysis. Significantly enriched (FDR<0.05) gene ontology terms (GOTERM) and pathways (KEGG_PATHWAY) were identified with DAVID (http://david.abcc.ncifcrf.gov/). The columns in the table contain information from the DAVID Functional Annotation Chart Report: Category (source provenance for the Term); Term (gene set name); Count (number of genes associated with this gene set); P-value (modified Fisher Exact P-value); List Total (number of genes in your query list mapped to any gene set in this ontology); Pop Hits (number of genes annotated to this gene set on the background list); Pop Total (number of genes on the background list mapped to any gene set in this ontology); and false discovery rate (FDR).
